# Immune Response to Tick-Borne Hemoparasites: Host Adaptive Immune Response Mechanisms as Potential Targets for Therapies and Vaccines

**DOI:** 10.3390/ijms21228813

**Published:** 2020-11-20

**Authors:** Alessandra Torina, Valeria Blanda, Sara Villari, Antonio Piazza, Francesco La Russa, Francesca Grippi, Marco Pio La Manna, Diana Di Liberto, José de la Fuente, Guido Sireci

**Affiliations:** 1 Area Diagnostica Sierologica, Istituto Zooprofilattico Sperimentale della Sicilia, via Gino Marinuzzi 3, 90129 Palermo, Italy; alessandra.torina@izssicilia.it (A.T.); francesca.grippi@izssicilia.it (F.G.); 2 Laboratorio di Riferimento OIE Theileriosi, Istituto Zooprofilattico Sperimentale della Sicilia, via Gino Marinuzzi 3, 90129 Palermo, Italy; 3 Laboratorio di Entomologia e Controllo Vettori Ambientali, Istituto Zooprofilattico Sperimentale della Sicilia, Via Gino Marinuzzi 3, 90129 Palermo, Italy; sara.villari@izssicilia.it (S.V.); antoniopiazza.vet@gmail.com (A.P.); francesco.larussa@izssicilia.it (F.L.R.); 4 Central Laboratory of Advanced Diagnostic and Biological Research (CLADIBIOR), BIND, University Hospital “Paolo Giaccone”, Università degli studi di Palermo, Via del Vespro 129, 90100 Palermo, Italy; marcopio.lamanna@unipa.it (M.P.L.M.); diana.diliberto@unipa.it (D.D.L.); guido.sireci@unipa.it (G.S.); 5 SaBio, Instituto de Investigación en Recursos Cinegéticos IREC-CSIC-UCLM-JCCM, Ronda de Toledo s/n, 13005 Ciudad Real, Spain; josedejesus.fuente@uclm.es; 6 Department of Veterinary Pathobiology, Center for Veterinary Health Sciences, Oklahoma State University, Stillwater, OK 74078, USA

**Keywords:** tick-borne hemoparasites, adaptive immune response, antigens

## Abstract

Tick-transmitted pathogens cause infectious diseases in both humans and animals. Different types of adaptive immune mechanisms could be induced in hosts by these microorganisms, triggered either directly by pathogen antigens or indirectly through soluble factors, such as cytokines and/or chemokines, secreted by host cells as response. Adaptive immunity effectors, such as antibody secretion and cytotoxic and/or T helper cell responses, are mainly involved in the late and long-lasting protective immune response. Proteins and/or epitopes derived from pathogens and tick vectors have been isolated and characterized for the immune response induced in different hosts. This review was focused on the interactions between tick-borne pathogenic hemoparasites and different host effector mechanisms of T- and/or B cell-mediated adaptive immunity, describing the efforts to define immunodominant proteins or epitopes for vaccine development and/or immunotherapeutic purposes. A better understanding of these mechanisms of host immunity could lead to the assessment of possible new immunotherapies for these pathogens as well as to the prediction of possible new candidate vaccine antigens.

## 1. Tick-Borne Hemoparasites and Interactions with Hosts

Ticks are considered to be second, worldwide, to mosquitoes as vectors of human diseases, but they are the most important vectors of disease-causing pathogens in domestic and wild animals [1,2]. Awareness on the impacts of tick-borne diseases is increasing [3], and they constitute a significant number of emerging infectious diseases [4].

The family Anaplasmataceae (order Rickettsiales) contains the tick-transmitted genera *Anaplasma* and *Ehrlichia*, infectious agents of companion, domestic and wildlife species, as well as of humans [5].

*Anaplasma* spp. is an obligate intracellular pathogen living inside host cells, protected from the host immune system. *Anaplasma marginale*, one of the most widespread pathogens of this genus, is the aetiologic agent of cattle anaplasmosis, a disease severely affecting the cattle industry [6]. The inflammatory disease results from the harmful response elicited by the host and it includes fever, anemia, weight loss, abortion, reduced milk production and, sometimes, death [7]. The zoonotic agent *Anaplasma phagocytophilum*, differently from *A. marginale*, does not replicate in erythrocytes but in circulating mature granulocytes and endothelial cells, causing high fever and leukopenia [8].

*Ehrlichia* spp. is an animal and human pathogen with public health importance [9]. The main *Ehrlichia* species with recognized zoonotic potential are *Ehrlichia chaffeensis* and *Ehrlichia ewingii* [10]. *Ehrlichia* species infect endothelial cells and white blood cells [10] and are able to survive in phagocytes, evading the immune response of the host and reprogramming the host cell defense mechanisms [11,12]. *E. chaffeensis,* one of the most investigated species, especially infects monocytes/macrophages, where it resides in an early endosome. *E. chaffeensis* survives in the host cell by inhibiting the fusion of phagosome and lysosome to evade destruction by lysosomal enzymes [13]. Both an excessive immune response against *E. chaffeensis* as well as a weak response in immunocompromised patients lead to a severe disease.

*Rickettsia* genus (family Rickettsiaceae, order Rickettsiales) includes an expanding number of species differing in antigenic and microbiological characteristics, ecology, distribution pathogenicity and association with arthropod hosts. *Rickettsia* species are traditionally classified into the Spotted Fever Group and the Typhus Group, with most of the known species belonging to the former [14]. At the site of arthropod inoculation, a localized rickettsial infection may be present as an eschar (“tache noir”). Following this, endothelial cells represent the primary targets for rickettsia infection since one of the main pathologic effects of rickettsial infection is increased vascular permeability. Disseminated infection may result in severe vasculitis and endothelial damage [15].

Among tick-borne protozoa, piroplasms of *Babesia* and *Theileria* genera are widespread pathogens causing economic losses worldwide. *Babesia* spp. (order Piroplasmida, family Babesidae) infects and multiplies inside erythrocytes, resulting in red blood cell lysis [16]. More than one hundred *Babesia* species exist and are able to infect a wide range of vertebrate hosts. In particular, cattle babesiosis, mainly due to *Babesia bovis* and *Babesia bigemina*, causes relevant economic losses in several countries. Infections with *Babesia* are also common in wild animals, although usually subclinical [17]. Babesiosis is also a zoonosis of increasing importance [18,19]. Infected hosts are able to develop immunity towards *Babesia* species, involving both humoral and cellular factors [16].

*Theileria* spp. (order Piroplasmida, family Theileridae) infects leukocytes at the sporozoite stage. Inside leucocytes, sporozoites multiply by merogony and then schizonts develop in merozoites that are released and invade red blood cells, forming piroplasms [20]. *Theileria* species infect domestic and wild animals; they can be gathered into schizont “transforming” or “non-transforming” species. Transforming parasites include species responsible for severe disease—among these are *Theileria annulata* (agent of tropical theileriosis) and *Theileria parva* (agent of East Coast fever) in cattle and *Theileria lestoquardi* (agent of malignant theileriosis) in small ruminants [21].

Non-transforming species, i.e., *Theileria orientalis, Theileria mutans* and *Theileria velifera*, are usually considered as benign but they also include pathogenic species [22]. *Theileria* parasites develop within the cytoplasm of host leukocytes, where the endosomal cell membrane dissolves, making the parasite not accessible to antibodies.

The comprehension of tick–host–pathogen interactions at the cellular and molecular levels, as, for example, the mechanisms regarding the immune response elicited in the host by the pathogen, is an essential issue for characterizing pathogen transmission, establishment and pathogenesis and for identifying novel checkpoints for pathogen control [2]. This review examines the interactions of the above-mentioned pathogens with different effector mechanisms of T- and/or B cell-mediated adaptive immunity, describing the efforts to define immunodominant proteins or epitopes for vaccine development and/or immunotherapeutic purposes.

## 2. Adaptive Immune Response to Antigens Derived from Tick-Transmitted Hemoparasites: A Useful Tool to Analyze Immunogenicity of Molecules

### 2.1. Anaplasma spp.

The outer membrane fraction of *A. marginale* is composed of at least six major surface polypeptides, which include the major surface proteins (MSPs) MSP-1a, MSP-1b, MSP-2, MSP-3, MSP-4 and MSP-5. Immunization with purified *A. marginale* outer membranes can induce complete protection against infection by homologous strains, probably due to CD4^+^ T-lymphocyte-mediated Interferon gamma (IFN-γ) release and secretion of immunoglobulin G (IgG)-2 antibodies against outer membrane protein epitopes. Protection against homologous challenge was shown in cattle immunized with purified *A. marginale* native MSP-1, MSP-2 and MSP-3, with significant reductions in anemia [23].

Recombinant proteins could be used as subunit vaccines to reduce the high costs of outer membrane purification and many types of nanoparticles have been already explored as nano-carriers for improving their immunogenicity. As an example, Pimentel and colleagues recently used carbon nanotubes as antigen delivery systems, taking advantage of nanotubes’ ability to protect the attached molecules against enzymatic degradation and to efficiently cross biological membranes [24]. The nanocomplex included the core motif of *A. marginale* MSP1a adsorbed onto the nanoparticle surface of a carbon nanotube. When the nanocomplex was used for mice immunization, it stimulated the production of IgG1 and IgG2a and increased the expression of pro-inflammatory (Tumor necrosis factor (TNF)-α; interleukin (IL)-12 and IL-18) and anti-inflammatory (IL-10) cytokines. Compared to the immunization with peptide-adjuvant, a balanced Th1 and Th2 immune response was induced.

Even a subcutaneous ear implant vaccine was developed for a slow release of the vaccine construct. *A. marginale* MSP1a was administered subcutaneously as priming, while a bio-erodible polyanhydride rod with intermediate slow release was used for boosting and the ear implant deposited subcutaneously was used to release antigen for a long time. The subcutaneous construct induced a durable protection against the pathogen, in particular when multiple adjuvants were used for antigen delivery [25].

Alternative subunit vaccines for *A. marginale* would include the use of subdominant proteins present in the outer membrane, showing the vantage of not changing during the infection and being highly conserved among different strains. They include the proteins of *A. marginale* type IV secretion system, a 1.05-MDa complex that spans the outer and inner bacterial membranes, whose role is mainly the host cell adhesion/invasion, and the elongation factor-Tu (Ef-Tu), a membrane-associated protein belonging to the family of hydrolases involved in protein synthesis [26]. Two other proteins of the *A. marginale* type IV secretion system, VirB9.1 and VirB9.2 induced a humoral and cellular immunity, stimulating CD4^+^ T-lymphocyte proliferation, IFN-γ secretion and IgG2 production in outer membrane-immunized cattle [27]. However, the vaccine based on the recombinant proteins VirB9.1, VirB9.2, VirB10, VirB11 and Ef-Tu was able to elicit high IgG2 titers in the immunized calves but failed to protect against *A. marginale* challenge. All the animals showed clinical symptoms and required treatments, suggesting that further studies are required to identify and express, in the native form, the main epitopes of these antigens [26].

*A. phagocytophilum* infection induces a mild immune suppression in humans and mice and the control is mediated in a CD4^+^ T cell-dependent mechanism. IFN-γ both aids in pathogen clearance and induces host pathology [28]. As for the other pathogens, the challenge is to find vaccine antigens able to cross-protect from many strains. The immunization carried out using the whole bacterium, indeed, did not protect the animals from an *A. phagocytophilum* infection [29].

### 2.2. Ehrlichia spp.

The role of CD4^+^ T cells in *Ehrlichia* infections has not yet been clarified as CD4 knockout (KO) mice have been reported to be capable of resolving *E. chaffeensis* experimental infection [30]. However, resolution of infection is increased or more rapid in the presence of CD4^+^ T cells both for *E. chaffeensis* and for *E. ruminantium* [31]. CD4^+^ T cells in *Ehrlichia* infection have shown to produce IFN-γ; in fact, IFN-γ KO mice were very susceptible to infection [32]. Interestingly, a combined depletion of IFN-γ and TNF-α showed a further detrimental effect on infection survival [33], suggesting a controlled interaction between such mediators in *Ehrlichia* infection.

In order to identify CD4^+^ Th1 epitopes inducing IFN-γ production, a screening of ten *E. ruminantium* proteins (Erum0660, Erum1150, Erum2540, Erum5420, Erum7140, Erum7320, Erum7350, Erum7360, Erum7620 and Erum8010) previously identified as immunogenic was carried out to determine their ability to induce cellular immunity in sheep [34]. The authors identified 23 peptides stimulating different cell-mediated immune responses. These peptides could efficiently induce memory CD4^+^ T cells to rapidly proliferate and significantly increase IFN-γ production in immune sheep Peripheral Blood Mononuclear Cells (PBMCs). The antigens represent possible vaccine candidates for inclusion in a multi-epitope vaccine that could overcome many limitations of the live blood vaccine for *E. ruminantium*, so far the only commercially available vaccine for this pathogen.

CD8^+^ T cells seem to be involved in the cross-regulation of IL-10 production during *Ehrlichia* infection. TNF-α promotes IL-10 production, which downregulates TNF-α expression [35]. The major role of CD8^+^ in the form of cytotoxic T-lymphocyte activity and/or gamma interferon production in experimental *E. chaffeensis* and *E. muris* infection remains to be elucidated [33]. In any case, CD8^+^ T cell-mediated tissue damage is related to fatal murine ehrlichiosis in association with TNF-α and IL-10 overproduction and CD4^+^ Th1 hyporesponsiveness [36].

Other *Ehrlichia* immune-reactive identified antigens include ferric ion-binding protein, disulfide bond formation protein, ankyrin repeat proteins (i.e., *E. canis* gp200), and tandem repeat proteins (TRP-) [37,38,39,40,41,42,43]. Several orthologs of TRPs have been discovered in *E. chaffeensis* and *E. canis*—for example, TRP120/TRP140, TRP75/TRP95, TRP47/TRP36 and TRP32/TRP19 [40]. Through interaction with several host proteins, TRPs contribute to the infection establishment. Antibodies against the most important TRPs in *E. chaffeensis* showed both in vitro inhibition of replication and in vivo reduction in bacterial load [44].

A recently identified *E. muris* TRP, P29, recognized as the ortholog *E. chaffeensis* TRP47 and *E. canis* TRP36, was found to generate a strong IgG antibody response and to stimulate CD4^+^ T cell differentiation into IFN-γ-producing Th1 effector/memory cells, activating macrophage microbicide activity. These effects suggest a possible inclusion for *E. muris* P29 in a subunit vaccine against ehrlichiosis [40].

Although antibodies are not generally effective during intracellular infections, Winslow and colleagues [45] reported an antibody role in *E. chaffeensis* infection control that was observed in both normal mice and mice with severe combined immunodeficiency (SCID). In particular, antibodies, usually IgG2, directed towards the bacterial Outer Membrane Proteins (OMPs), showed an elevated affinity and a long binding half-life. Probably, these antibodies stimulated the infected macrophages to kill and eliminate the phagocytized bacteria, inducing the expression by infected macrophages of different effector molecules, as inflammatory cytokines, reactive nitrogen intermediates, chemokines [46,47,48] by activating Fc Gamma Receptors. A second proposed action mechanism implies a role of the antibodies in bacteria opsonization during intercellular transfer, when the pathogen is exposed to their action.

Antibodies against *Ehrlichia*, OMPs P28-9 and P28-19 and heat-shock protein 60 were found to induce protection in mice [49,50]. Human monoclonal antibodies against *E. chaffeensis* OMP-1 were able to inhibit infection through both extracellular and intracellular effector mechanisms [51]. In particular, the methods proposed by the authors include a novel intracellular mechanism mediated by the tripartite motif protein 21 (TRIM21). The complex antibody *E. chaffeensis,* sensed by TRIM21, was able to initiate a significant pro-inflammatory response and recruit autophagic regulators and effectors, leading to rapid degradation of *E. chaffeensis* by selective autophagy. The results showed that humoral immunity in humans is effective against the intracellular pathogen *E. chaffeensis*, suggesting that these anti-*E. chaffeensis* human monoclonal antibodies could be potential candidates for immunotherapy.

A relevant role of liver and spleen as generative sites of B cell responses to *E. muris* in wild-type C57BL/6 carrier mice was recently investigated [52]. The authors found a proliferation of B cells within infected livers. Moreover, the occurrence of somatic hypermutation of B-cell clones was revealed by high-throughput sequencing of V regions, showing clonal mixing and trafficking between spleen and liver.

Regarding vaccination, no approved vaccine against *E. chaffeensis* infection is currently available. McGill and colleagues [53] recently proposed a potential vaccine candidate, showing that an attenuated mutant strain of *E. chaffeensis*, reporting a mutation in the Ech_0660 gene, was able to induce protection in dogs. The effect was observed not only when wild-type *E. chaffeensis* was intravenously administered [54] but also when the pathogen was tick-transmitted [53]. Ech_0660-vaccinated dogs produced antibodies against *E. chaffeensis,* and strong CD4^+^T cell responses and released a high IL-17 production, suggesting a possible role for IL-17 in the immune response to ehrlichial pathogens.

In a following study [55], the ability to protect from wild-type *E. chaffeensis* of another mutant organism, Ech_0230, was investigated, presenting defects in the capacity to replicate in vivo in the vertebrate host. However, unlike the Ech_0660 mutant, Ech_0230 vaccination with this second mutant did not protect the animals from subsequent wild-type challenge, probably due to the transient induced IgG response. The authors described, for the first time, in dogs infected with *E. chaffeensis*, a significant expansion of CD4^+^CD8^+^ double-positive T cells, whose role in immunity to persistent *E. chaffeensis* infection is not understood. This population undergoes clonal expansion and is involved in IFN-γ and IL-17 secretion and in the increase of granulysin and granzyme B production in response to *E. chaffeensis* antigen.

### 2.3. Rickettsia spp.

Studies on adaptive immunity and rickettsial infections have been closely related to scientific efforts for the identification of vaccine candidates, especially of T cell antigens potentially conferring cross-protection against different *Rickettsia* species.

Accumulating evidence in recent years has demonstrated a leading role of T cells in protection from *Rickettsia* infection, mainly via CD8^+^ T cells-mediated bacteria killing and CD4^+^ T cells that sustain protective immune reaction releasing effector factors [56]. As concerning CD8^+^ T cells, studies in experimental infections have proven that granzyme B enhanced expression, as well as IFN-γ production by CD8^+^ cells, induce *Rickettsia* killing [57]. Interestingly, CD8^+^-associated cytotoxic activity seems to be of evident importance in *R. australis* experimental infection but not in *R. typhi* infection [58]. Such an observation remarks that although cytotoxic activity is essential for *Rickettsia* killing, IFN-γ production by CD8^+^ cells is a relevant event contributing to limiting the infection from some *Rickettsia* species, as demonstrated in studies involving IFN-γ KO mice as CD8^+^ donors [59]. CD4^+^ T cells positively influence the activity of phagocytic cells in rickettsial infection [60]; major cytokines produced by activated CD4^+^ T cells in bacterial infections have been reported to be IFN-γ and TNF-α, related to a potentiated activity on nitric oxide-mediated killing by macrophages. However, it is worthy to note that CD4^+^ T cells may also develop in Th17 lineage, with production of IL-17A and IL-22 responsible for downstream regulation of a range of target cells [57]. Indeed, apart from stimulating nitric oxide (NO) production by macrophages, activated by IFN-γ, they orchestrate the promotion of further cytokine release from target tissue cells and immune phagocytic cells, thus sustaining the inflammatory environment for rickettsial killing. As for humoral immunity, the contribution of antibodies (Abs) on defense against rickettsial infection is still under investigation. The effects of immunization with Abs against rickettsia outer membrane proteins A and B have been evaluated in an experimental model of *R. conorii* infection, conferring protection mainly via opsonization mechanisms [61]. However, given the development of such Abs in a late infection phase, raising about 10–12 days after experimental infection, a possible involvement in secondary infections has been proposed. Instead, Ab anti-lipopolysaccharides appeared up to day 6 but their role and contribution in protection against rickettsia have been not established yet [62].

Since Rickettsiae need to proficiently exploit metabolite synthesis from host cells due to their condition of obligate intracellular bacteria, their effective survival strictly depends on successful invasion of host cells. Thus, *Rickettsia* surface cell antigens (sca) have been extensively recognized as key factors in adherence and invasion of target cells as endothelial and phagocytic cells. Sca family comprises 17 genes encoding proteins with a modular structure including an N-terminal domain, a central passenger domain and a C-terminal domain [63]; within sca family, five genes are conserved especially between Spotted Fever-Group Rickettsiae: sca0 (rOmpA), sca1, sca2, sca4 and sca5 (rOmpB). Apart from the demonstration of the protective action of Ab anti-OmpA or Ab anti-OmpB against *R. conorii* infection in experimental mice model [61], OmpB protein-derived synthetic peptides have been reported to positively influence CD8^+^ T cells, suggesting their potential in supporting adaptive immunity for vaccination purposes [64]. Moreover, the genes codifying these omp proteins contain both regions highly conserved among the different *Rickettsia* species as well as hypervariable regions, and for this reason, they are considered attractive targets for molecular detection and differentiation [65]. Other studies demonstrated the immune recognition of other Scas, as Sca1, Sca2 and Sca4 [63], although the contribution of each such protein in comparison with the most studied OmpA and OmpB still need to be established.

In an interesting study by Caro-Gomez et al. [66], a reverse vaccinology approach allowed for identifying potential antigens able to induce CD8^+^ T cells, CD4^+^ T cells or both. The *R. prowazekii* ORFeome has been analyzed in silico, identifying 100 and 85 rickettsial proteins displaying high-affinity binding peptides for Major Histocompatibility Complex (MHC) class I or class II (for CD8^+^ and CD4^+^ T cells activation, respectively); interestingly, 21 proteins contained peptides binding both to MHC classes I and II, suggesting their potential as vaccine candidates, by triggering both CD4^+^ and CD8^+^ T cell responses. Using an experimental mice model, it has been reported that five antigens (RP739, RP403, RP734, RP598 and RP778) conferred protection against *R. typhi* infection in mice via the stimulation of CD8^+^ T cells and IFN-γ-related production. Of interest, RP778 also promoted CD4^+^ T cells’ IFN-γ production, suggesting its ability to stimulate both CD4^+^ and CD8^+^ T cells. Although candidate antigens have been selected from the *R. prowazekii* ORFeome, protection was afforded towards *R. typhi*, suggesting a cross-protection between different *Rickettsia* species belonging to the Typhus Group. Furthermore, Caro-Gomez et al. [66] tested the potential of the same antigens to induce protection against *R. conorii* infection, resulting in 62.5% of mice being protected, in line with the protein similarity of ortholog antigens between *R. prowazekii* and *R. conorii* (73–90%). In addition, given that Rickettsiae have been also recognized as potential bioweapons, the definition of a successful vaccination strategy may represent an obligate challenge in the forthcoming years [67].

### 2.4. Babesia spp.

Host susceptibility to develop babesiosis has been related to genetic traits, as few murine strains (mainly C3H/HeJ) are susceptible to *Babesia* infections. Genetic tolerance was shown by Duangijnda et al. [68], who found increased frequencies of bovine leukocyte antigens (BoLA) in animals infected by *Babesia* spp. and *A. marginale*.

Cytokines may modulate the pathogenicity of *Babesia* spp. as, for example, IFN-γ production was not found in PBMC supernatant from *Babesia* sp. *BQ1* (Lintan)-infected sheep. On the contrary, IFN-γ production was detected in *B. divergens*-infected sheep. The inverse occurred for IL-10, suggesting an important role for IFN-γ and IL-10 in sheep infections caused by these two pathogens. Indeed, IFN-γ produced during *B. divergens* infection may have a protective role, explaining the weak infectivity of *B. divergens* in sheep. In contrast, IL-10 was produced early during *Babesia* spp. BQ1 (Lintan) infection, and this could be responsible for the absence of IFN-γ production and the higher pathogenicity of this *Babesia* species in sheep [69].

Immunity derived by spleen cells was analyzed for its role in resolution of *B. microti* infection. Mice experimentally infected with *B. microti* showed significant splenic B- and T cell depletion and an increase in macrophage levels, suggesting their relevancy for disease resolution. Infected mice also showed significantly higher plasmatic concentration of CD4+ Th1 cells-secreted cytokines, such as IL-2 and IFN-γ. Thus, Th1 cell-mediated immunity appears to be important in the clearance of this intracellular pathogen. A significant increase was measured for IL-6, promoting differentiation of Th17 cells, while only moderate variations were observed for other Th17 cell-produced cytokines, such as IL-17A, IL-17F, IL-21 and IL-22 [70].

As concerning *Babesia* spp. immunogenic proteins, it was reported that *B. bovis* small heat shock protein (Hsp20) is recognized by CD4^+^ T lymphocytes from cattle that recovered from infection and immune to challenge. The protein shows a predicted amino acid sequence conserved among different *B. bovis* strains. Immune cattle were stimulated with truncated recombinant Hsp20 and overlapping peptides to define the location of CD4^+^ T-cell epitopes for possible inclusion in vaccines. Both amino-terminal (amino acids 1 to 105) and carboxyterminal (amino acids 48 to 177) regions resulted immunogenic, stimulating a strong T-cell proliferation and IFN-γ production. Moreover, amino acids from 11 to 62 of Hsp20 induced a T-cell response in animals with different MHC haplotypes [71].

Another investigated protein, *B. bovis* Rhoptry-associated protein 1 (RAP-1), is recognized by B- and T lymphocytes from cattle that recovered from infection and were immune to subsequent challenge and it could represent a possible vaccine candidate for *B. bovis* and *B. bigemina* bovine infections. Immunization with either the native or recombinant protein reduced parasitemia in challenged animals. An N-terminal portion of *B. bovis* RAP-1 containing immunodominant T-cell epitopes was used as a vaccine in combination with IL-12 and RIBI adjuvant to induce a type 1 response, in order to verify if it would prime calves for antibody and T-helper cell responses. RAP-1-specific IgG titers, T-lymphocyte proliferation and IFN-γ production were observed following inoculations of either recombinant full-length or the N-terminal portion of RAP-1. However, in spite of the presence of strong RAP-1-specific IgG and CD4^+^ T-lymphocyte responses, neither antigen stimulated a protective immune response [72].

The major surface antigen of *B. microti* merozoites (BMSA) was also evaluated as a potential vaccine candidate. The BMSA-induced immune response significantly inhibited pathogen invasion of the host erythrocytes and in vivo parasite growth. The protection was associated to the increase in the Th17 cytokine IL-17, the Th1 cytokine IL-12p70 and Th2 cytokines, such as IL-4 and IL-10. Ingenuity pathway analysis showed that IL-17 facilitated the secretion of Th2 cytokines, stimulating a Th2 response and promoting the high level of IgG1 expression. Furthermore, anti-BMSA monoclonal antibodies were able to confer protection to NOD/SCID mice from a challenge with *B. microti* [73].

Apical Membrane Antigen-1 of *Babesia divergens* (BdAMA-1) has been also analyzed as a vaccine candidate by evaluating its polymorphism and studying the humoral response against BdAMA-1 in sheep experimentally infected with *B. divergens*. Two BdAMA-1 haplotypes (A and B) were defined based on two nonsynonymous point mutations. Prediction of linear epitopes showed very similar antigenicity of the two haplotypes. Antibody production against the extracellular domain of BdAMA-1 is weak and late and it includes both IgG1 and IgG2. These results indicate that even if BdAMA-1 may not be an immunodominant antigen, it could induce a mixed type 1 and type 2 immune response [74]. AMA-1 orthologs have been also reported in other *Babesia* species [75,76,77,78].

AMA-1 proteins interact with the rhoptry neck protein 2 (RON2), which integrates to the red blood cell membrane after its secretion from the rhoptries in a complex formed with other RON proteins. For such reasons, a possible role of vaccine candidate for RON2 in *Babesia* species has been evaluated and a highly conserved gene encoding for RON2 was reported in *B. bovis* [79]. In this study, the authors designed and chemically synthesized four conserved peptides, containing predicted B-cell epitopes in hydrophilic regions of this protein. Antibodies anti-RON2 generated towards these peptides were able to recognize intraerythrocytic merozoites of *B. bovis*. Two of these peptides were also able to induce partially neutralizing antibodies that blocked the invasion process, as observed in the in vitro neutralization assays. Even in *B. bigemina* [80], RON2 is expressed in merozoites and contains conserved B-cell epitopes inducing neutralizing antibodies in naturally infected cattle.

A recombinant modified vaccinia virus Ankara vector expressing a chimeric multi-antigen comprehending Merozoite Surface Antigen—(MSA) 2c, RAP-1 and Hsp20 was obtained and evaluated as a vaccine candidate [81]. The chimeric multi-antigen comprises immunodominant B- and T cell regions of three *B. bovis* proteins and it was used to immunize mice in homologous and heterologous prime-boost with a recombinant protein cocktail. The best results were obtained by combining a prime of a protein cocktail and a boost with the recombinant virus. This protocol induced a high level of specific IgG antibodies and secreted IFN-γ and activation of IFN-γ^+^-, CD4^+^- and CD8^+^-specific T cells. In a following study, it was investigated if the use of adenoviral vectors as antibody-triggering agents would increase humoral immune responses. Results in mice confirmed that adenoviruses, as well as the bacterially expressed multi-antigen, are highly reliable primer candidates, inducing high percentages of CD4^+^ and CD8^+^ T cells with a Th1 cytokine profile [82]. Subsequently, the same authors [83] used this subunit vaccine as a prime to immunize 13–15-month-old Holstein-Friesian steers with a modified vaccinia Ankara vector as a boost. Both prime and boost expressed a chimeric multi-antigen including the immunodominant B and T cell epitopes of the three aforementioned *B. bovis* proteins (MSA–2c, RAP-1 and hsp20). Response to the new vaccine was compared with the one to *B. bovis* live attenuated vaccine used in Argentina (R1A). After the immunization, all bovines were inoculated with a virulent *B. bovis* strain. Both groups of vaccinated animals developed high titers of total IgG antibodies and an antigen-specific Th1 cellular response before and after the challenge. However, steers immunized with the subunit vaccine showed clinical signs of disease, indicating a lack of protection with this recombinant formulation and that further improvements are needed to achieve the desired effectiveness.

As concerning the role of B cells, it was reported that following *B. microti* infection, B cells secrete IL-10 and that naive B cells stimulated in vitro with *B. microti* produced high amounts of IL-10 [84]. This study showed that B cells sorted from *B. microti*-infected mice produced higher IL-10 levels in response to LPS stimulation than B cells sorted from naive mice. B cell-deficient mice developed significantly lower levels of parasitemia after *B. microti* infection and exhibited lower levels of serum IL-10 compared to wild-type mice, suggesting that IL-10-producing B cells may play a role in susceptibility to *B. microti*. These IL-10-producing B cells, also known as Bregs, were identified in experimental models of autoimmune diseases, cancer and helminth infections. In the same study, it was observed that the number of IL-10-producing CD1d high CD5^+^ Bregs significantly increased during the acute phase of *B. microti* infection, suggesting that these cells have a critical role in the susceptibility to the pathogen. *B. microti*-induced Bregs are associated with the induction of CD4^+^CD25^+^ FoxP3^+^ regulatory t-cells, also known as Tregs. More specifically, the frequency of CD4 ^+^CD25^+^FoxP3^+^ Tregs increased in concomitance with the IL-10-producing Breg development, while Treg induction by *B. microti* infection did not occur in the absence of IL-10-producing Bregs. These observations suggest that the anti-infective immunity could modulate many immunopathologies through different mechanisms comprising induction of T- and/or B regulatory cells [85].

### 2.5. Theileria spp.

The intracytoplasmic location favors cell-mediated immune responses and, in particular, immune recognition by CD8^+^ T cells of parasite proteins released into the host cell and exposed through the class I MHC. Many *Theileria* species are able to, in vitro, transform mononuclear leukocytes into continuously dividing cells, expressing surface MHC classes I and II [86] that have been extensively used in studies of CD8^+^ T cell responses [87]. Nevertheless, how they kill parasitized cells is still poorly known. As already reported for human and murine CD8^+^ T cells, and also in bovine, probably, CD8^+^ T cells act through granule exocytosis and, specifically, granzyme B [88]. Cytotoxic T lymphocytes (CTLs) usually elicit responses restricted to the parasite strain and this is related to the issue of cross-protection towards different strains.

Studies about antigenic variability among *T. parva* strains suggest that such cross-protection is incomplete [89]. For this reason, a combination of parasites derived from three *T. parva* isolates, known as the “Muguga cocktail”, is administered to confer a broader protection. However, some findings showed no substantial differences in CTL response following immunization with only the Muguga strain in comparison with the cocktail [90]. The analysis of CTL responses in animals of the same MHC I haplotype immunized with the Muguga cocktail showed a differential recognition of autologous cells even between haploidentical animals.

The preferential induction of responses to particular antigens is due to the presence of T cells with high-avidity receptors for the respective epitopes [91,92]. However, in purified populations of bovine naive CD8^+^ T cells cocultured with autologous *T. parva*-infected lymphoblasts, a specific CTL activity was observed only in the presence of specific CD4^+^ T cells. The presence of non-specific CD4^+^ T cells or cytokine preparations maintained the helper function only for immune, not naive, CTL cells [93].

Possible vaccine candidate antigens specific for CD8^+^ T cells (Table 1) have been identified in *T. parva* [94,95,96,97] and nine promising epitopes in six *T. parva* antigens were identified, together with their respective BoLA MHC class I restriction elements [92]. Among these antigens, Tp1 and Tp2 are hypothetical proteins of unknown function, Tp7 is an Hsp90, Tp4 is the ε subunit of T-complex protein 1 and Tp5 and Tp8 have been identified as the elongation translation initiation factor 1A and a cysteine protease, respectively. Some of these antigens, in particular in Tp1 and Tp2, show a polymorphic variability [98,99,100]. Tp1 inoculated in cattle promoted survival at lethal doses of the pathogen and increased percentages of memory lymphocytes, confirming a promising role of this antigen for subunit vaccine development [101].

Animals subjected to repeated *T. parva* sporozoite challenge showed antibodies able to neutralize sporozoites in vitro [117], with most of them recognizing the sporozoite surface protein p67 of *T. parva* [102]. Subunit vaccination using a recombinant p67 elicited levels of protection up to 70% [118].

The polymorphic immunodominant molecule (PIM) expressed in *T. parva* sporozoites and schizonts is a target for murine monoclonal antibodies with neutralizing activity [103]. PIM causes a strong humoral response and is employed in a diagnostic ELISA [104].

Other *T. parva* antigens include the p150 antigen and the major merozoite/piroplasm surface antigen of 32 kDa. The first, a polymorphic antigen with variability in a region of repeated sequences, is recognized by recovery sera, expressed in sporozoite microspheres and, at low levels, in schizonts [119]. The second one induces high titers of specific antibodies in cattle but fails in conferring protection against the pathogen [120].

*T. annulata* elicits different adaptive immune responses [121,122] and stimulates cytokine release to favor presentation of parasite antigens to CD4^+^ T cells. These cells produce IFN-γ that acts on non-infected macrophages, activating their production of TNF-α and nitric oxide (NO), which destroy the infected cells. CD8^+^ T cells kill infected leukocytes, recognizing parasite MHC-presented antigens. The clinical signs and pathological lesions of the disease are caused by overproduction of cytokines [123], dissemination of transformed macrophages through the lymphatic system and other organs [124] and a dysregulated immune response [125].

B cells are arrested in the draining lymph nodes and do not differentiate, probably due to the elevated levels of IFN-γ. This inhibition may be a pathogen mechanism to change the uncontrolled Th1 responses observed both in vivo and in vitro [106,126].

*T. annulata*-infected macrophages acquire aberrant phenotype and show dendritic cell features, including enhanced antigen-presenting capabilities [127,128,129]. Moreover, the pathogen polyclonally activates naive T cells in a non-specific, superantigen manner [106,126] with loss of CD2 expression on the T cell surface [130], disrupting the normal T lymphocyte response and subsequent protective immunity [131]. Such a process is considered as the *T. annulata* immune evasion strategy. Tolerant cattle show less lymph node enlargement following *T. annulata* infection, related to a decreased proliferation of macrophages and T lymphocytes and the development of an appropriate immune response and recovery [132,133]. CD4^+^ T cell activation preceded that of CD8^+^ T cells, which shows a biphasic trend, with the first phase directed against MHC antigens of the immunizing cell line, and the second one, MHC class I-restricted, associated with a parasite-specific immune response [134]. For example, WC1^+^ gamma/delta TcR^+^ T cells recognize a surface determinant on *T. annulata*-infected cells but they need a second signal to proliferate, which can be provided by exogenous IL-2. Infected cells express cytokine transcripts encoding IL-1 alpha, IL-1 beta, IL-6 and TNFα, as well as IL-10, IL-12, IL-15 and type I interferons, but not IFN-γ, IL-2, IL-4 and IL-7 [106,135]. Among interferons, only type I IFN, in particular IFN-β, is expressed [86].

*T. annulata* antigens Ta5 and Ta9, the orthologs of *T. parva* Tp5 and Tp9, are recognized by CD8^+^ T cells, while a third antigen, not previously identified in *T. parva*, has been named Ta11 [105]. Another highly investigated *T. annulata* candidate is the major surface sporozoite antigen, SPAG-1, a 92-kDa protein containing elastin repeats. Antibodies towards its C-terminus inhibit sporozoite entry, even if SPAG-1-specific T cell lines were IL-2 dependent and produced IL-4 but not IFN-γ, suggesting that the induced immune response may require additional factors to be effective [106]. The major merozoite piroplasm surface antigen, a 32-kDa antigen also known as Tams1, is a prominent serological determinant of *T. annulata*, highly polymorphic among and within parasite isolates [107].

Both SPAG-1 and Tams1 confer partial protection using different delivery systems and adjuvants, but their antigenic diversity in parasite strains restrict their effectiveness as vaccine candidates [136].

A *T. annulata* surface protein (TaSP) is recognized by sera of cattle that recovered from infection. The protein, considered the *T. annulata* homologue of *T. parva* PIM, is expressed in the sporozoite and schizont stages [108].

*T. orientalis* do not transform leukocytes and the pathogenic phase is due to erythrocyte destruction and anemia. The immune response is largely cell-mediated, and the role of the humoral response is not very clear [137]. Moreover, although enlarged cells containing schizont-like structures have been identified in infected cattle [138], details of schizont development are unclear [139]. The spleen shows a relevant role in the acute infection [140] and it may be also involved in the serological response via the white pulp [137]. It is unknown whether acutely *T. orientalis*-infected animals become refractory to subsequent disease.

*T. orientalis* major piroplasm surface protein (MPSP), the ortholog of *T. parva* p32 and *T. annulata* Tams-1 [109], is highly expressed during both the sporozoite [110] and piroplasm phases [111,137] and it includes determinants for humoral and cellular immunity [112,113]. The IgG response to this protein correlated to clinical signs, parasite genotype and infection intensity [138]. This antigen has also been considered as a vaccine candidate [112] and two CD4^+^ T cell epitopes were detected showing amino acid substitutions among field variants [113]. Despite high antibody titers against MPSP in persistently infected cattle, host humoral immunity against MPSP is insufficient to completely control the infection [112] and, furthermore, antibody response against MPSP can be type-specific [114,115].

A *T. orientalis* gene with weak homology to Tp9/Ta9 was reported but its role in CTL response has not been clarified yet [109].

As concerns *T. lestoquardi,* mechanisms involved in protective immunity against the pathogen are still unknown [141,142]. Two *T. lestoquardi* proteins, Tl8 (putative cysteine proteinase, 349 aa) and Tl9 (hypothetical secreted protein, 293 aa), orthologs of *T. parva* and *T. annulata* genes, were identified as possible vaccine candidates. These antigens are recognized by in vitro CD8^+^ T cell lines, suggesting a possible involvement in protective T cell responses in vivo against *T. lestoquardi*-infected leukocytes [116].

## 3. Conclusions

Host immune response could interact with *Babesia* spp., *Rickettsia* spp., *Anaplasma* spp., *Erlichia* spp. and *Theileria* spp. using different cells and/or molecules responsible for innate or adaptive immunity. The protective effects on infectious diseases by different branches of immunity are related to the phases of the infection. Effector mechanisms of innate immune responses, rapidly displayed by the host, are able to inhibit the symptoms of the acute phase of infections [143], while the T- and B cell responses, mounted by the immune system after a while, are able to induce a long-term protection against pathologies induced by hemoparasites. Many different molecules are able to induce T- and/or B cell responses and could be considered as candidate vaccine antigens (Table 1). However, as recently proposed, the combination of tick-derived and pathogen-derived antigens would result in more effective and safe vaccines [144,145,146]. To further advance in this direction, the quantum vaccinomics approach based on the combination of protective epitopes or immunological quantum will allow for the design of regional and host/tick species-driven interventions for vaccine formulation and implementation [147,148]. Intriguingly, tick-transmitted pathogens could exert the modulation of autoimmune as well as allergic disorders, as demonstrated in different models of helminth infections. In conclusion, the immune response to pathogens transmitted by tick bites activates various effector mechanisms of innate and adaptive immunity, able to induce different types of protective or deleterious side effects for the host, such as autoimmune or allergic disorders. Different immunization routes and vaccine formulations offer promising perspectives for the control of tick-borne diseases while reducing side effects promoted by protective immunity.

## Figures and Tables

**Table 1:** Overview of the main candidate vaccine antigens with the related immune response induced for each pathogen.

Pathogen	Protein	Immune Response Induced	References
*Anaplasma* spp.	Native Major Surface Protein-1 (MSP-1), MSP-2 and MSP-3 outer membranes	CD4+ T lymphocyte and Immunoglobulin G2 responses	[23]
	*A. marginale* MSP1a + carbon nanotubes	Production of IgG1 and IgG2a, increases in pro-TNF-α, IL-12, IL-18 and IL-10 cytokines	[24]
	*A. marginale* type IV secretion system proteins, VirB9-1 and VirB9-2	CD4^+^ T-lymphocyte proliferation and IgG2 production	[27]
*Ehrlichia* spp.	*E. ruminantium* peptides from Erum0660, Erum1150, Erum2540, Erum5420, Erum7140. Erum7320, Erum7350, Erum7360, Erum7620 and Erum8010	Stimulation of memory CD4^+^ T cells proliferation and IFN-γ production in immune sheep Peripheral blood mononuclear cell (PBMCs)	[34]
	Outer Membrane Proteins (OMPs) - P28 (OMP-1) - P28-9 (OMP-1g) - P28-12 - P28-19	- Specific IgG2a release - Protection in vivo	[49,50,51]
	Tandem Repeat Proteins (TRPs) - *E. chaffensis/E. canis* orthologs RP120/TRP140, TRP75/TRP95, TRP47/TRP36 and TRP32/TRP19	- Epitope-specific antibody (Ab) release, mainly IgG1 - Inhibition of Signal transducer and activator of transcription 1 (Stat1), Janus Kinase 1 and 2 (Jak1 and Jak2) phosphorylation induced by IFN- γ (for TRP47) - Replication inhibition in vitro - Reduction in bacterial load in vivo	[37,38,39,40,41,42,43,44]
	- *E. muris* ortholog P29	- Strong IgG antibody response - IFN-γ expression in CD4+ T cells, activating macrophage microbicidal activity	[40]
	Ankyrin repeat proteins - E. chaffeensis Ank200	- Specific antibodies release - TNF-α induction - Modulation of expression of Jak/Stat genes	[42]
	Heat-shock protein 60 (Hsp60/GroEL)	- Specific antibodies release - Protection in vivo	[50]
	- Ferric ion-binding protein (Fbp)	- Specific antibodies release	[38]
*Rickettsia* spp.	Surface Cell Antigens (Sca) - OmpA - OmpB	- Production of opsonising Abs promoting phagocyte-dependent killing of Rickettsiae - Protection from experimental *R. conorii* infection	[61,63,64]
	Candidate antigens from *R. prowazekii* - RP739 - RP403 - RP734 - RP598 - RP778	- Development of CD8^+^ T cells expressing IFN-γ - Protection from experimental *R. typhi* and *R. conorii* infection (cross-protection)	[66]
	RP778	- IFN-γ expression in CD4^+^ T cells	[66]
	RP778	- IFN-γ expression in CD4^+^ T cells	[66]
*Babesia* spp.	Rhoptry-Associated Protein (RAP-1)	Antigen-specific IgG and IFN-γ release	[72]
	Heat-shock protein 20 (Hsp 20)	T cell proliferation and IFN-γ production	[71]
	*B. microti* surface antigen (BMSA)	Th17 and Th2 activation and IgG1 secretion	[73]
	*B. divergens* Apical Membrane Antigen-1 (BdAMA-1)	IgG1, IgG2, Th1 and Th2 activation	[74]
	*B. bovis* rhoptry neck protein 2 (RON2)	Induction of partially neutralizing antibodies that blocked the invasion process	[79]
	Chimeric multi-antigen	Antigen-specific IgG1, CD4^+^ and CD8^+^ IFN-γ^+^	[81,82,83]
*Theileria parva*	Tp1–Tp2–Tp4–Tp5–Tp7–Tp8	CD8^+^ T-cell responses	[92,94,95,96,97,98,99,100,101]
	p67	Ab production	[102]
	Polymorphyc immunodominant molecule (PIM)	Production of Ab with neutralizing activity	[103,104]
	p150	Ab production	
*Theileria annulata*	Ta5–Ta9–Ta11	CD8^+^ T-cell responses	[105]
	SPAG-1	Induction of IL-4-producing T-cells	[106]
	Tams1	Ab production	[107]
	TaSP (homologue of *T. parva* PIM)	Production of neutralizing Ab	[108]
*Theileria orientalis*	Major piroplasm surface protein (MPSP)	Ab production	[109,110,111,112,113,114,115]
*Theileria lestoquardi*	Tl8 –Tl9	CD8^+^ T-cell responses	[116]

## References

[1] De la Fuente J., Estrada-Pena A., Venzal J.M., Kocan K.M., Sonenshine D.E. (2008). Overview: Ticks as vectors of pathogens that cause disease in humans and animals. Front. Biosci..

[2] Wikel S.K. (2018). Ticks and Tick-Borne Infections: Complex Ecology, Agents, and Host Interactions. Vet. Sci..

[3] Dantas-Torres F., Chomel B.B., Otranto D. (2012). Ticks and Tick-Borne Diseases: A One Health Perspective. Trends Parasitol..

[4] Jones K.E., Patel N.G., Levy M.A., Storeygard A., Balk D., Gittleman J.L., Daszak P. (2008). Global Trends in Emerging Infectious Diseases. Nature.

[5] Rar V., Golovljova I. (2011). Anaplasma, Ehrlichia, and “Candidatus Neoehrlichia” Bacteria: Pathogenicity, Biodiversity, and Molecular Genetic Characteristics, a Review. Infect. Genet. Evol..

[6] Naranjo V., Ruiz-Fons F., Höfle U., Fernández de Mera I.G., Villanúa D., Almazán C., Torina A., Caracappa S., Kocan K.M., Gortázar C. (2006). Molecular epidemiology of human and bovine anaplasmosis in southern Europe. Ann. N. Y. Acad. Sci..

[7] Aubry P., Geale D.W. (2011). A Review of Bovine Anaplasmosis. Transbound. Emerg. Dis..

[8] Woldehiwet Z. (2006). *Anaplasma phagocytophilum* in Ruminants in Europe. Ann. N. Y. Acad. Sci..

[9] Ismail N., McBride J.W. (2017). Tick-Borne Emerging Infections: Ehrlichiosis and Anaplasmosis. Clin. Lab. Med..

[10] Moumène A., Meyer D.F. (2016). Ehrlichia’s Molecular Tricks to Manipulate Their Host Cells. Microbes Infect..

[11] McBride J.W., Walker D.H. (2011). Molecular and Cellular Pathobiology of Ehrlichia Infection: Targets for New Therapeutics and Immunomodulation Strategies. Expert Rev. Mol. Med..

[12] Lina T.T., Farris T., Luo T., Mitra S., Zhu B., McBride J.W. (2016). Hacker within! Ehrlichia Chaffeensis Effector Driven Phagocyte Reprogramming Strategy. Front. Cell. Infect. Microbiol..

[13] Rikihisa Y. (2010). Anaplasma Phagocytophilum and Ehrlichia Chaffeensis: Subversive Manipulators of Host Cells. Nat. Rev. Microbiol..

[14] Scarpulla M., Barlozzari G., Marcario A., Salvato L., Blanda V., De Liberato C., D’Agostini C., Torina A., Macrì G. (2016). Molecular detection and characterization of spotted fever group rickettsiae in ticks from Central Italy. Ticks Tick Borne Dis..

[15] Sahni S.K., Rydkina E. (2009). Host-Cell Interactions with Pathogenic Rickettsia Species. Future Microbiol..

[16] Homer M.J., Aguilar-Delfin I., Telford S.R., Krause P.J., Persing D.H. (2000). Babesiosis. Clin. Microbiol. Rev..

[17] Beugnet F., Moreau Y. (2015). Babesiosis. Rev. Sci. Tech..

[18] Vannier E.G., Diuk-Wasser M.A., Ben Mamoun C., Krause P.J. (2015). Babesiosis. Infect. Dis. Clin. N. Am..

[19] Krause P.J. (2019). Human Babesiosis. Int. J. Parasitol..

[20] Mans B.J., Pienaar R., Latif A.A. (2015). A Review of Theileria Diagnostics and Epidemiology. Int. J. Parasitol. Parasites Wildl..

[21] Dobbelaere D., Heussler V. (1999). Transformation of Leukocytes by Theileria Parva and T. Annulata. Annu. Rev. Microbiol..

[22] Sivakumar T., Hayashida K., Sugimoto C., Yokoyama N. (2014). Evolution and Genetic Diversity of Theileria. Infect. Genet. Evol..

[23] Brown W.C., Shkap V., Zhu D., McGuire T.C., Tuo W., McElwain T.F., Palmer G.H. (1998). CD4^+^ T-Lymphocyte and Immunoglobulin G2 Responses in Calves Immunized with *Anaplasma marginale* Outer Membranes and Protected against Homologous Challenge. Infect. Immun..

[24] Pimentel L.S., Turini C.A., Santos P.S., de Morais M.A., Souza A.G., Barbosa M.B., do Nascimento Martins E.M., Coutinho L.B., Furtado C.A., Ladeira L.O. (2020). Balanced Th1/Th2 Immune Response Induced by MSP1a Functional Motif Coupled to Multiwalled Carbon Nanotubes as Anti-Anaplasmosis Vaccine in Murine Model. Nanomed. Nanotechnol. Biol. Med..

[25] Curtis A.K., Reif K.E., Kleinhenz M.D., Martin M.S., Skinner B., Kelly S.M., Jones D.E., Reppert E.J., Montgomery S.R., Narasimhan B. (2019). Development of a Subcutaneous Ear Implant to Deliver an Anaplasmosis Vaccine to Dairy Steers. J. Anim. Sci..

[26] Sarli M., Novoa M.B., Mazzucco M.N., Signorini M.L., Echaide I.E., de Echaide S.T., Primo M.E. (2020). A Vaccine Using *Anaplasma marginale* Subdominant Type IV Secretion System Recombinant Proteins Was Not Protective against a Virulent Challenge. PLoS ONE.

[27] Zhao L., Mahony D., Cavallaro A.S., Zhang B., Zhang J., Deringer J.R., Zhao C.-X., Brown W.C., Yu C., Mitter N. (2016). Immunogenicity of Outer Membrane Proteins VirB9-1 and VirB9-2, a Novel Nanovaccine against *Anaplasma marginale*. PLoS ONE.

[28] Brown W.C. (2012). Adaptive Immunity to Anaplasma Pathogens and Immune Dysregulation: Implications for Bacterial Persistence. Comp. Immunol. Microbiol. Infect. Dis..

[29] Stuen S., Okstad W., Artursson K., Al-Khedery B., Barbet A., Granquist E.G. (2015). Lambs Immunized with an Inactivated Variant of Anaplasma Phagocytophilum. Acta Vet. Scand..

[30] Ganta R.R., Cheng C., Wilkerson M.J., Chapes S.K. (2004). Delayed Clearance of Ehrlichia Chaffeensis Infection in CD4+ T-Cell Knockout Mice. Infect. Immun..

[31] Mwangi D.M., McKeever D.J., Nyanjui J.K., Barbet A.F., Mahan S.M. (2002). Immunisation of Cattle against Heartwater by Infection with Cowdria Ruminantium Elicits T Lymphocytes That Recognise Major Antigenic Proteins 1 and 2 of the Agent. Vet. Immunol. Immunopathol..

[32] Bitsaktsis C., Huntington J., Winslow G. (2004). Production of IFN-Gamma by CD4 T Cells Is Essential for Resolving Ehrlichia Infection. J. Immunol..

[33] Feng H.-M., Walker D.H. (2004). Mechanisms of Immunity to Ehrlichia Muris: A Model of Monocytotropic Ehrlichiosis. Infect. Immun..

[34] Thema N., Tshilwane S.I., Pretorius A., Son L., Smith R.M., Steyn H.C., Liebenberg J., van Kleef M. (2019). Identification and Characterisation of Conserved Epitopes of E. Ruminantium That Activate Th1 CD4^+^ T Cells: Towards the Development of a Multi-Epitope Vaccine. Mol. Immunol..

[35] Ismail N., Soong L., McBride J.W., Valbuena G., Olano J.P., Feng H.-M., Walker D.H. (2004). Overproduction of TNF-α by CD8^+^ Type 1 Cells and Down-Regulation of IFN-γ Production by CD4^+^ Th1 Cells Contribute to Toxic Shock-Like Syndrome in an Animal Model of Fatal Monocytotropic Ehrlichiosis. J. Immunol..

[36] Stevenson H.L., Estes M.D., Thirumalapura N.R., Walker D.H., Ismail N. (2010). Natural Killer Cells Promote Tissue Injury and Systemic Inflammatory Responses During Fatal Ehrlichia-Induced Toxic Shock-Like Syndrome. Am. J. Pathol..

[37] Ohashi N., Unver A., Zhi N., Rikihisa Y. (1998). Cloning and Characterization of Multigenes Encoding the Immunodominant 30-Kilodalton Major Outer Membrane Proteins of Ehrlichia Canis and Application of the Recombinant Protein for Serodiagnosis. J. Clin. Microbiol..

[38] Doyle C.K., Zhang X., Popov V.L., McBride J.W. (2005). An Immunoreactive 38-Kilodalton Protein of Ehrlichia Canis Shares Structural Homology and Iron-Binding Capacity with the Ferric Ion-Binding Protein Family. Infect. Immun..

[39] McBride J.W., Ndip L.M., Popov V.L., Walker D.H. (2002). Identification and Functional Analysis of an Immunoreactive DsbA-Like Thio-Disulfide Oxidoreductase of Ehrlichia spp.. Infect. Immun..

[40] Thirumalapura N.R., Crocquet-Valdes P.A., Saito T.B., Thomas S., McBride J.W., Walker D.H. (2013). Recombinant Ehrlichia P29 Protein Induces a Protective Immune Response in a Mouse Model of Ehrlichiosis. Vaccine.

[41] Luo T., Zhang X., Wakeel A., Popov V.L., McBride J.W. (2008). A Variable-Length PCR Target Protein of Ehrlichia Chaffeensis Contains Major Species-Specific Antibody Epitopes in Acidic Serine-Rich Tandem Repeats. Infect. Immun..

[42] Luo T., Zhang X., Nicholson W.L., Zhu B., McBride J.W. (2010). Molecular Characterization of Antibody Epitopes of Ehrlichia Chaffeensis Ankyrin Protein 200 and Tandem Repeat Protein 47 and Evaluation of Synthetic Immunodeterminants for Serodiagnosis of Human Monocytotropic Ehrlichiosis. Clin. Vaccine Immunol..

[43] McBride J.W., Zhang X., Wakeel A., Kuriakose J.A. (2011). Tyrosine-Phosphorylated Ehrlichia Chaffeensis and Ehrlichia Canis Tandem Repeat Orthologs Contain a Major Continuous Cross-Reactive Antibody Epitope in Lysine-Rich Repeats. Infect. Immun..

[44] Kuriakose J.A., Zhang X., Luo T., McBride J.W. (2012). Molecular Basis of Antibody Mediated Immunity against Ehrlichia Chaffeensis Involves Species-Specific Linear Epitopes in Tandem Repeat Proteins. Microbes Infect..

[45] WINSLOW G.M., YAGER E., LI J.S.-Y. (2003). Mechanisms of Humoral Immunity during Ehrlichia Chaffeensis Infection. Ann. N. Y. Acad. Sci..

[46] Bayón Y., Alonso A., Sánchez Crespo M. (1997). Stimulation of Fc Gamma Receptors in Rat Peritoneal Macrophages Induces the Expression of Nitric Oxide Synthase and Chemokines by Mechanisms Showing Different Sensitivities to Antioxidants and Nitric Oxide Donors. J. Immunol..

[47] Marsh C.B., Anderson C.L., Lowe M.P., Wewers M.D. (1996). Monocyte IL-8 Release Is Induced by Two Independent Fc Gamma R- Mediated Pathways. J. Immunol..

[48] Marsh C.B., Wewers M.D., Tan L.C., Rovin B.H. (1997). Fc(Gamma) Receptor Cross-Linking Induces Peripheral Blood Mononuclear Cell Monocyte Chemoattractant Protein-1 Expression: Role of Lymphocyte Fc(Gamma)RIII. J. Immunol..

[49] Crocquet-Valdes P.A., Thirumalapura N.R., Ismail N., Yu X., Saito T.B., Stevenson H.L., Pietzsch C.A., Thomas S., Walker D.H. (2011). Immunization with Ehrlichia P28 Outer Membrane Proteins Confers Protection in a Mouse Model of Ehrlichiosis. Clin. Vaccine Immunol..

[50] Thomas S., Thirumalapura N.R., Crocquet-Valdes P.A., Luxon B.A., Walker D.H. (2011). Structure-Based Vaccines Provide Protection in a Mouse Model of Ehrlichiosis. PLoS ONE.

[51] Velayutham T.S., Kumar S., Zhang X., Kose N., Walker D.H., Winslow G., Crowe J.E., McBride J.W. (2019). Ehrlichia Chaffeensis Outer Membrane Protein 1-Specific Human Antibody-Mediated Immunity Is Defined by Intracellular TRIM21-Dependent Innate Immune Activation and Extracellular Neutralization. Infect. Immun..

[52] Trivedi N., Weisel F., Smita S., Joachim S., Kader M., Radhakrishnan A., Clouser C., Rosenfeld A.M., Chikina M., Vigneault F. (2019). Liver Is a Generative Site for the B Cell Response to Ehrlichia Muris. Immunity.

[53] McGill J.L., Nair A.D.S., Cheng C., Rusk R.A., Jaworski D.C., Ganta R.R. (2016). Vaccination with an Attenuated Mutant of Ehrlichia Chaffeensis Induces Pathogen-Specific CD4^+^ T Cell Immunity and Protection from Tick-Transmitted Wild-Type Challenge in the Canine Host. PLoS ONE.

[54] Nair A.D.S., Cheng C., Jaworski D.C., Ganta S., Sanderson M.W., Ganta R.R. (2015). Attenuated Mutants of Ehrlichia Chaffeensis Induce Protection against Wild-Type Infection Challenge in the Reservoir Host and in an Incidental Host. Infect. Immun..

[55] McGill J.L., Wang Y., Ganta C.K., Boorgula G.D.Y., Ganta R.R. (2018). Antigen-Specific CD4^+^CD8^+^ Double-Positive T Cells Are Increased in the Blood and Spleen During Ehrlichia Chaffeensis Infection in the Canine Host. Front. Immunol..

[56] Valbuena G., Jordan J.M., Walker D.H. (2004). T Cells Mediate Cross-Protective Immunity between Spotted Fever Group Rickettsiae and Typhus Group Rickettsiae. J. Infect. Dis..

[57] Moderzynski K., Heine L., Rauch J., Papp S., Kuehl S., Richardt U., Fleischer B., Osterloh A. (2017). Cytotoxic effector functions of T cells are not required for protective immunity against fatal Rickettsia typhi infection in a murine model of infection: Role of TH1 and TH17 cytokines in protection and pathology. PLoS Negl. Trop. Dis..

[58] Moderzynski K., Papp S., Rauch J., Heine L., Kuehl S., Richardt U., Fleischer B., Osterloh A. (2016). CD4+ T Cells Are as Protective as CD8+ T Cells against Rickettsia Typhi Infection by Activating Macrophage Bactericidal Activity. PLoS Negl. Trop. Dis..

[59] Walker D.H., Olano J.P., Feng H.-M. (2001). Critical Role of Cytotoxic T Lymphocytes in Immune Clearance of Rickettsial Infection. Infect. Immun..

[60] Fang R., Ismail N., Soong L., Popov V.L., Whitworth T., Bouyer D.H., Walker D.H. (2007). Differential Interaction of Dendritic Cells with Rickettsia Conorii: Impact on Host Susceptibility to Murine Spotted Fever Rickettsiosis. Infect. Immun..

[61] Feng H.-M., Whitworth T., Popov V., Walker D.H. (2004). Effect of Antibody on the Rickettsia-Host Cell Interaction. Infect. Immun..

[62] Feng H.-M., Whitworth T., Olano J.P., Popov V.L., Walker D.H. (2004). Fc-Dependent Polyclonal Antibodies and Antibodies to Outer Membrane Proteins A and B, but Not to Lipopolysaccharide, Protect SCID Mice against Fatal Rickettsia Conorii Infection. Infect. Immun..

[63] Blanc G., Ngwamidiba M., Ogata H., Fournier P.E., Claverie J.M., Raoult D. (2005). Molecular evolution of rickettsia surface antigens: Evidence of positive selection. Mol. Biol. Evol..

[64] Li Z., Díaz-Montero C.M., Valbuena G., Yu X.-J., Olano J.P., Feng H.-M., Walker D.H. (2003). Identification of CD8 T-Lymphocyte Epitopes in OmpB of Rickettsia Conorii. Infect. Immun..

[65] Blanda V., D’Agostino R., Giudice E., Randazzo K., La Russa F., Villari S., Vullo S., Torina A. (2020). New Real-Time PCRs to Differentiate Rickettsia spp. and Rickettsia conorii. Molecules.

[66] Caro-Gomez E., Gazi M., Goez Y., Valbuena G. (2014). Discovery of Novel Cross-Protective Rickettsia Prowazekii T-Cell Antigens Using a Combined Reverse Vaccinology and in Vivo Screening Approach. Vaccine.

[67] Azad A.F. (2007). Pathogenic Rickettsiae as Bioterrorism Agents. Clin. Infect. Dis..

[68] Duangjinda M., Jindatajak Y., Tipvong W., Sriwarothai J., Pattarajinda V., Katawatin S., Boonkum W. (2013). Association of BoLA-DRB3 Alleles with Tick-Borne Disease Tolerance in Dairy Cattle in a Tropical Environment. Vet. Parasitol..

[69] Guan G., Chauvin A., Yin H., Luo J., Moreau E. (2010). Course of Infection by Babesia Sp. BQ1 (Lintan) and B. Divergens in Sheep Depends on the Production of IFNγ and IL10. Parasite Immunol..

[70] Djokic V., Akoolo L., Parveen N. (2018). Babesia Microti Infection Changes Host Spleen Architecture and Is Cleared by a Th1 Immune Response. Front. Microbiol..

[71] Norimine J., Mosqueda J., Palmer G.H., Lewin H.A., Brown W.C. (2004). Conservation of Babesia Bovis Small Heat Shock Protein (Hsp20) among Strains and Definition of T Helper Cell Epitopes Recognized by Cattle with Diverse Major Histocompatibility Complex Class II Haplotypes. Infect. Immun..

[72] Norimine J., Mosqueda J., Suarez C., Palmer G.H., McElwain T.F., Mbassa G., Brown W.C. (2003). Stimulation of T-Helper Cell Gamma Interferon and Immunoglobulin G Responses Specific for Babesia Bovis Rhoptry-Associated Protein 1 (RAP-1) or a RAP-1 Protein Lacking the Carboxy-Terminal Repeat Region Is Insufficient To Provide Protective Immunity against Virulent B. Bovis Challenge. Infect. Immun..

[73] Man S., Fu Y., Guan Y., Feng M., Qiao K., Li X., Gao H., Cheng X. (2017). Evaluation of a Major Surface Antigen of Babesia Microti Merozoites as a Vaccine Candidate against Babesia Infection. Front. Microbiol..

[74] Moreau E., Bonsergent C., Al Dybiat I., Gonzalez L.M., Lobo C.A., Montero E., Malandrin L. (2015). Babesia Divergens Apical Membrane Antigen-1 (BdAMA-1): A Poorly Polymorphic Protein That Induces a Weak and Late Immune Response. Exp. Parasitol..

[75] Gaffar F.R., Yatsuda A.P., Franssen F.F.J., de Vries E. (2004). Erythrocyte Invasion by Babesia Bovis Merozoites Is Inhibited by Polyclonal Antisera Directed against Peptides Derived from a Homologue of Plasmodium Falciparum Apical Membrane Antigen 1. Infect. Immun..

[76] Zhou J., Yang J., Zhang G., Nishikawa Y., Fujisaki K., Xuan X. (2006). Babesia Gibsoni: An Apical Membrane Antigen-1 Homologue and Its Antibody Response in the Infected Dogs. Exp. Parasitol..

[77] Torina A., Agnone A., Sireci G., Mosqueda J.J., Blanda V., Albanese I., La Farina M., Cerrone A., Cusumano F., Caracappa S. (2010). Characterization of the Apical Membrane Antigen-1 in Italian Strains of Babesia Bigemina. Transbound. Emerg. Dis..

[78] Torina A., Cordaro A., Blanda V., D’Agostino R., Scimeca S., Scariano M.E., Sireci G., Lelli R. (2016). A promising new ELISA diagnostic test for cattle babesiosis based on *Babesia bigemina* Apical Membrane Antigen-1. Vet. Ital..

[79] Hidalgo-Ruiz M., Suarez C.E., Mercado-Uriostegui M.A., Hernandez-Ortiz R., Ramos J.A., Galindo-Velasco E., León-Ávila G., Hernández J.M., Mosqueda J. (2018). Babesia Bovis RON2 Contains Conserved B-Cell Epitopes That Induce an Invasion-Blocking Humoral Immune Response in Immunized Cattle. Parasites Vectors.

[80] Mosqueda J., Hidalgo-Ruiz M., Calvo-Olvera D.A., Hernandez-Silva D.J., Ueti M.W., Mercado-Uriostegui M.A., Rodriguez A., Ramos-Aragon J.A., Hernandez-Ortiz R., Kawazu S. (2019). RON2, a Novel Gene in Babesia Bigemina, Contains Conserved, Immunodominant B-Cell Epitopes That Induce Antibodies That Block Merozoite Invasion. Parasitology.

[81] Jaramillo Ortiz J.M., Del Médico Zajac M.P., Zanetti F.A., Molinari M.P., Gravisaco M.J., Calamante G., Wilkowsky S.E. (2014). Vaccine Strategies against Babesia Bovis Based on Prime-Boost Immunizations in Mice with Modified Vaccinia Ankara Vector and Recombinant Proteins. Vaccine.

[82] Jaramillo Ortiz J.M., Molinari M.P., Gravisaco M.J., Paoletta M.S., Montenegro V.N., Wilkowsky S.E. (2016). Evaluation of Different Heterologous Prime-Boost Immunization Strategies against Babesia Bovis Using Viral Vectored and Protein-Adjuvant Vaccines Based on a Chimeric Multi-Antigen. Vaccine.

[83] Jaramillo Ortiz J.M., Paoletta M.S., Gravisaco M.J., López Arias L.S., Montenegro V.N., de la Fournière S.A.M., Valenzano M.N., Guillemi E.C., Valentini B., Echaide I. (2019). Immunisation of Cattle against Babesia Bovis Combining a Multi-Epitope Modified Vaccinia Ankara Virus and a Recombinant Protein Induce Strong Th1 Cell Responses but Fails to Trigger Neutralising Antibodies Required for Protection. Ticks Tick Borne Dis..

[84] Jeong Y.-I., Hong S.-H., Cho S.-H., Lee W.-J., Lee S.-E. (2012). Induction of IL-10-Producing CD1dhighCD5^+^ Regulatory B Cells Following Babesia Microti-Infection. PLoS ONE.

[85] Maizels R.M., McSorley H.J. (2016). Regulation of the Host Immune System by Helminth Parasites. J. Allergy Clin. Immunol..

[86] Sager H., Brunschwiler C., Jungi T.W. (1998). Interferon Production by Theileria Annulata-Transformed Cell Lines Is Restricted to the Beta Family. Parasite Immunol..

[87] Hart J., MacHugh N.D., Morrison W.I. (2011). Theileria Annulata-Transformed Cell Lines Are Efficient Antigen-Presenting Cells for in Vitro Analysis of CD8 T Cell Responses to Bovine Herpesvirus-1. Vet. Res..

[88] Yang J., Pemberton A., Morrison W.I., Connelley T. (2018). Granzyme B Is an Essential Mediator in CD8+ T Cell Killing of Theileria Parva-Infected Cells. Infect. Immun..

[89] Radley D.E., Brown C.G.D., Cunningham M.P., Kimber C.D., Musisi F.L., Payne R.C., Purnell R.E., Stagg S.M., Young A.S. (1975). Chemoprophylactic immunization of cattle against Theileria parva (Muguga) and five Theileria strains. Vet. Parasitol..

[90] Steinaa L., Svitek N., Awino E., Njoroge T., Saya R., Morrison I., Toye P. (2018). Immunization with One Theileria Parva Strain Results in Similar Level of CTL Strain-Specificity and Protection Compared to Immunization with the Three-Component Muguga Cocktail in MHC-Matched Animals. BMC Vet. Res..

[91] Morrison W.I. (1996). Influence of Host and Parasite Genotypes on Immunological Control of Theileria Parasites. Parasitology.

[92] Graham S.P., Pellé R., Yamage M., Mwangi D.M., Honda Y., Mwakubambanya R.S., de Villiers E.P., Abuya E., Awino E., Gachanja J. (2008). Characterization of the Fine Specificity of Bovine CD8 T-Cell Responses to Defined Antigens from the Protozoan Parasite Theileria Parva. Infect. Immun..

[93] Taracha E.L., Awino E., McKeever D.J. (1997). Distinct CD4^+^ T Cell Helper Requirements in Theileria Parva-Immune and -Naive Bovine CTL Precursors. J. Immunol..

[94] Hemmink J.D., Weir W., MacHugh N.D., Graham S.P., Patel E., Paxton E., Shiels B., Toye P.G., Morrison W.I., Pelle R. (2016). Limited Genetic and Antigenic Diversity within Parasite Isolates Used in a Live Vaccine against Theileria Parva. Int. J. Parasitol..

[95] Graham S.P., Pellé R., Honda Y., Mwangi D.M., Tonukari N.J., Yamage M., Glew E.J., de Villiers E.P., Shah T., Bishop R. (2006). Theileria Parva Candidate Vaccine Antigens Recognized by Immune Bovine Cytotoxic T Lymphocytes. Proc. Natl. Acad. Sci. USA.

[96] Goddeeris B.M., Morrison W.I., Toye P.G., Bishop R. (1990). Strain Specificity of Bovine Theileria Parva-Specific Cytotoxic T Cells Is Determined by the Phenotype of the Restricting Class I MHC. Immunology.

[97] Taracha E.L., Goddeeris B.M., Morzaria S.P., Morrison W.I. (1995). Parasite Strain Specificity of Precursor Cytotoxic T Cells in Individual Animals Correlates with Cross-Protection in Cattle Challenged with Theileria Parva. Infect. Immun..

[98] Katzer F., Ngugi D., Oura C., Bishop R.P., Taracha E.L.N., Walker A.R., McKeever D.J. (2006). Extensive Genotypic Diversity in a Recombining Population of the Apicomplexan Parasite Theileria Parva. Infect. Immun..

[99] Pelle R., Graham S.P., Njahira M.N., Osaso J., Saya R.M., Odongo D.O., Toye P.G., Spooner P.R., Musoke A.J., Mwangi D.M. (2011). Two Theileria Parva CD8 T Cell Antigen Genes Are More Variable in Buffalo than Cattle Parasites, but Differ in Pattern of Sequence Diversity. PLoS ONE.

[100] Kerario I.I., Chenyambuga S.W., Mwega E.D., Rukambile E., Simulundu E., Simuunza M.C. (2019). Diversity of Two Theileria Parva CD8+ Antigens in Cattle and Buffalo-Derived Parasites in Tanzania. Ticks Tick Borne Dis..

[101] Svitek N., Saya R., Awino E., Munyao S., Muriuki R., Njoroge T., Pellé R., Ndiwa N., Poole J., Gilbert S. (2018). An Ad/MVA Vectored Theileria Parva Antigen Induces Schizont-Specific CD8^+^ Central Memory T Cells and Confers Partial Protection against a Lethal Challenge. npj Vaccines.

[102] Dobbelaere D.A., Shapiro S.Z., Webster P. (1985). Identification of a Surface Antigen on Theileria Parva Sporozoites by Monoclonal Antibody. Proc. Natl. Acad. Sci. USA.

[103] Toye P., Musoke A., Naessens J. (2014). Role of the Polymorphic Immunodominant Molecule in Entry of Theileria Parva Sporozoites into Bovine Lymphocytes. Infect. Immun..

[104] Katende J., Morzaria S., Toye P., Skilton R., Nene V., Nkonge C., Musoke A. (1998). An Enzyme-Linked Immunosorbent Assay for Detection of Theileria Parva Antibodies in Cattle Using a Recombinant Polymorphic Immunodominant Molecule. Parasitol. Res..

[105] MacHugh N.D., Weir W., Burrells A., Lizundia R., Graham S.P., Taracha E.L., Shiels B.R., Langsley G., Morrison W.I. (2011). Extensive Polymorphism and Evidence of Immune Selection in a Highly Dominant Antigen Recognized by Bovine CD8 T Cells Specific for Theileria Annulata. Infect. Immun..

[106] Glass E.J. (2001). The Balance between Protective Immunity and Pathogenesis in Tropical Theileriosis: What We Need to Know to Design Effective Vaccines for the Future. Res. Vet. Sci..

[107] Dickson J., Shiels B.R. (1993). Antigenic Diversity of a Major Merozoite Surface Molecule in Theileria Annulata. Mol. Biochem. Parasitol..

[108] Schnittger L., Katzer F., Biermann R., Shayan P., Boguslawski K., McKellar S., Beyer D., Shiels B.R., Ahmed J.S. (2002). Characterization of a Polymorphic Theileria Annulata Surface Protein (TaSP) Closely Related to PIM of Theileria Parva: Implications for Use in Diagnostic Tests and Subunit Vaccines. Mol. Biochem. Parasitol..

[109] Hayashida K., Hara Y., Abe T., Yamasaki C., Toyoda A., Kosuge T., Suzuki Y., Sato Y., Kawashima S., Katayama T. (2012). Comparative Genome Analysis of Three Eukaryotic Parasites with Differing Abilities To Transform Leukocytes Reveals Key Mediators of Theileria-Induced Leukocyte Transformation. mBio.

[110] Sako Y., Sugimoto C., Onuma M. (1999). Expression of a Major Piroplasm Surface Protein of Theileria Sergenti in Sporozoite Stage. J. Vet. Med. Sci..

[111] Sugimoto C., Kawazu S., Kamio T., Fujisaki K. (1991). Protein Analysis of Theileria Sergenti/Buffeli/Orientalis Piroplasms by Two-Dimensional Polyacrylamide Gel Electrophoresis. Parasitology.

[112] Onuma M., Kakuda T., Sugimoto C. (1998). Theileria Parasite Infection in East Asia and Control of the Disease. Comp. Immunol. Microbiol. Infect. Dis..

[113] Kakuda T., Sugimoto C., Onuma M. (2001). Epitope-Mapping of Antigen-Specific T Lymphocyte in Cattle Immunized with Recombinant Major Piroplasm Surface Protein of Theileria Sergenti. J. Vet. Med. Sci..

[114] Iwasaki T., Kakuda T., Sako Y., Sugimoto C., Onuma M. (1998). Differentiation and Quantification of Theileria Sergenti Piroplasm Types Using Type-Specific Monoclonal Antibodies. J. Vet. Med. Sci..

[115] Yokoyama N., Sivakumar T., Ota N., Igarashi I., Nakamura Y., Yamashina H., Matsui S., Fukumoto N., Hata H., Kondo S. (2012). Genetic Diversity of Theileria Orientalis in Tick Vectors Detected in Hokkaido and Okinawa, Japan. Infect. Genet. Evol..

[116] Goh S., Ngugi D., Lizundia R., Hostettler I., Woods K., Ballingall K., MacHugh N.D., Morrison W.I., Weir W., Shiels B. (2016). Identification of Theileria Lestoquardi Antigens Recognized by CD8^+^ T Cells. PLoS ONE.

[117] Nene V., Morrison W.I. (2016). Approaches to Vaccination against Theileria Parva and Theileria Annulata. Parasite Immunol..

[118] Boulter N., Hall R. (1999). Immunity and Vaccine Development in the Bovine Theilerioses. Adv. Parasitol..

[119] Skilton R.A., Bishop R.P., Wells C.W., Spooner P.R., Gobright E., Nkonge C., Musoke A.J., Macklin M., Iams K.P. (1998). Cloning and Characterization of a 150 KDa Microsphere Antigen of Theileria Parva That Is Immunologically Cross-Reactive with the Polymorphic Immunodominant Molecule (PIM). Parasitology.

[120] Skilton R.A., Musoke A.J., Wells C.W., Yagi Y., Nene V., Spooner P.R., Gachanja J., Osaso J., Bishop R.P., Morzaria S.P. (2000). A 32 KDa Surface Antigen of Theileria Parva: Characterization and Immunization Studies. Parasitology.

[121] Preston P.M., Hall F.R., Glass E.J., Campbell J.D., Darghouth M.A., Ahmed J.S., Shiels B.R., Spooner R.L., Jongejan F., Brown C.G. (1999). Innate and Adaptive Immune Responses Co-Operate to Protect Cattle against Theileria Annulata. Parasitol. Today.

[122] Ferrolho J., Domingos A., Campino L. (2016). Cattle specific immune mechanisms used against the protozoan Theileria annulata. Int. Trends Immun..

[123] OIE (World Organization for Animal Health) (2015). Theileriosis. Manual of Diagnostic Tests and Vaccines for Terrestrial Animals.

[124] Forsyth L.M.G., Minns F.C., Kirvar E., Adamson R.E., Hall F.R., McOrist S., Brown C.G.D., Preston P.M. (1999). Tissue Damage in Cattle Infected WithTheileria AnnulataAccompanied by Metastasis of Cytokine-Producing, Schizont-Infected Mononuclear Phagocytes. J. Comp. Pathol..

[125] Brown D.J., Campbell J.D., Russell G.C., Hopkins J., Glass E.J. (1995). T Cell Activation by Theileria Annulata-Infected Macrophages Correlates with Cytokine Production. Clin. Exp. Immunol..

[126] Campbell J.D., Howie S.E., Odling K.A., Glass E.J. (1995). Theileria Annulata Induces Abberrant T Cell Activation in Vitro and in Vivo. Clin. Exp. Immunol..

[127] Morrison W.I., McKeever D.J. (2006). Current Status of Vaccine Development against Theileria Parasites. Parasitology.

[128] Glass E.J., Innes E.A., Spooner R.L., Brown C.G. (1989). Infection of Bovine Monocyte/Macrophage Populations with Theileria Annulata and Theileria Parva. Vet. Immunol. Immunopathol..

[129] Glass E.J., Spooner R.L. (1990). Parasite-Accessory Cell Interactions in Theileriosis. Antigen Presentation by Theileria Annulata-Infected Macrophages and Production of Continuously Growing Antigen-Presenting Cell Lines. Eur. J. Immunol..

[130] Nichani A.K., Craigmile S.C., Spooner R.L., Campbell J.D. (1999). Diminished IL-2 Responses and Alteration of CD2 Expression on CD8^+^ T Cells Are Associated with a Lack of Cytotoxic T Cell Responses during Theileria Annulata Infection. Clin. Exp. Immunol..

[131] Campbell J.D.M., Brown D.J., Nichani A.K., Howie S.E.M., Spooner R.L., Glass E.J. (1997). A Non-Protective T Helper 1 Response against the Intra-Macrophage Protozoan Theileria Annulata. Clin. Exp. Immunol..

[132] Glass E.J., Preston P.M., Springbett A., Craigmile S., Kirvar E., Wilkie G., Brown C.G.D. (2005). Bos Taurus and Bos Indicus (Sahiwal) Calves Respond Differently to Infection with Theileria Annulata and Produce Markedly Different Levels of Acute Phase Proteins. Int. J. Parasitol..

[133] Jensen K., Paxton E., Waddington D., Talbot R., Darghouth M.A., Glass E.J. (2008). Differences in the Transcriptional Responses Induced by Theileria Annulata Infection in Bovine Monocytes Derived from Resistant and Susceptible Cattle Breeds. Int. J. Parasitol..

[134] Nichani A.K., Campbell J.D.M., Glass E.J., Graham S.P., Craigmile S.C., Brown C.G.D., Spooner R.L. (2003). Characterization of Efferent Lymph Cells and Their Function Following Immunization of Cattle with an Allogenic Theileria Annulata Infected Cell Line. Vet. Immunol. Immunopathol..

[135] Collins R.A., Sopp P., Gelder K.I., Morrison W.I., Howard C.J. (1996). Bovine Gamma/Delta TcR^+^ T Lymphocytes Are Stimulated to Proliferate by Autologous Theileria Annulata-Infected Cells in the Presence of Interleukin-2. Scand. J. Immunol..

[136] Boulter N.R., Brown C.G., Kirvar E., Glass E., Campbell J., Morzaria S., Nene V., Musoke A., D’Oliveira C., Gubbels M.J. (1998). Different Vaccine Strategies Used to Protect against Theileria Annulata. Ann. N. Y. Acad. Sci..

[137] Jenkins C., Bogema D.R. (2016). Factors Associated with Seroconversion to the Major Piroplasm Surface Protein of the Bovine Haemoparasite Theileria Orientalis. Parasites Vectors.

[138] Sato M., Kamio T., Kawazu S., Taniguchi T., Minami T., Fujisaki K. (1993). Histological Observations on the Schizonts in Cattle Infected with Japanese Theileria Sergenti. J. Vet. Med. Sci..

[139] Sugimoto C., Fujisaki K., Dobbelaere D. (2002). Non-transforming Theileria parasites of ruminants. Theileria.

[140] Izzo M.M., Poe I., Horadagoda N., De Vos A.J., House J.K. (2010). Haemolytic Anaemia in Cattle in NSW Associated with Theileria Infections. Aust. Vet. J..

[141] El Imam A.H., Taha K.M. (2015). Malignant Ovine Theileriosis (Theileria lestoquardi): A Review. Jordan J. Biol. Sci..

[142] Leemans I., Fossum C., Johannisson A., Hooshmand-Rad P. (2001). Comparative Studies on Surface Phenotypes of Theileria Lestoquardi and T. Annulata Schizont-Infected Cells. Parasitol. Res..

[143] Torina A., Villari S., Blanda V., Vullo S., La Manna M.P., Shekarkar Azgomi M., Di Liberto D., de la Fuente J., Sireci G. (2020). Innate Immune Response to Tick-Borne Pathogens: Cellular and Molecular Mechanisms Induced in the Hosts. Int. J. Mol. Sci.

[144] De la Fuente J. (2018). Controlling Ticks and Tick-Borne Diseases…looking Forward. Ticks Tick Borne Dis..

[145] De La Fuente J., Villar M., Estrada-Peña A., Olivas J.A. (2018). High Throughput Discovery and Characterization of Tick and Pathogen Vaccine Protective Antigens Using Vaccinomics with Intelligent Big Data Analytic Techniques. Expert Rev. Vaccines.

[146] De la Fuente J., Estrada-Peña A. (2019). Why New Vaccines for the Control of Ectoparasite Vectors Have Not Been Registered and Commercialized?. Vaccines.

[147] De la Fuente J., Contreras M., Kasaija P.D., Gortazar C., Ruiz-Fons J.F., Mateo R., Kabi F. (2019). Towards a Multidisciplinary Approach to Improve Cattle Health and Production in Uganda. Vaccines.

[148] Artigas-Jerónimo S., Comín J.J.P., Villar M., Contreras M., Alberdi P., Viera I.L., Soto L., Cordero R., Valdés J.J., Cabezas-Cruz A. (2020). A Novel Combined Scientific and Artistic Approach for the Advanced Characterization of Interactomes: The Akirin/Subolesin Model. Vaccines.

